# A case of failed eradication of cystic fibrosis-related sinus colonisation by *Pseudomonas aeruginosa*

**DOI:** 10.1186/s12890-015-0113-0

**Published:** 2015-10-06

**Authors:** Barry Linnane, Linda Kearse, Nuala H. O’ Connell, John Fenton, Miranda G. Kiernan, Colum P. Dunne

**Affiliations:** Graduate Entry Medical School and Centre for Interventions in Infection, Inflammation & Immunity (4i), University of Limerick, Limerick, Ireland; University Hospital Limerick, Dooradoyle, Limerick, Ireland; National Children’s Research Centre (NCRC), SHIELD CF, Our Lady’s Children’s Hospital, Crumlin, Dublin, Ireland

**Keywords:** Cystic fibrosis, *Pseudomonas aeruginosa*, Paranasal sinuses, Eradication regimen

## Abstract

**Background:**

*Pseudomonas aeruginosa* is a pathogen associated with cystic fibrosis that has potential to decrease lung function and cause respiratory failure. Paranasal sinuses are increasingly recognised as potential reservoirs for intermittent colonisation by *P. aeruginosa*. This case documents investigation and outcome of *P. aeruginosa* recurrence in a male paediatric patient over an eight year period.

**Case presentation:**

A 12 year old Irish male paediatric cystic fibrosis patient experienced intermittent culturing of *P. aeruginosa* from the oropharyngeal region, indicating chronic infection of the sinuses despite absence of symptoms, retaining good lung function, and normal bronchoscopy and bronchoalveloar lavage. However, *P. aeruginosa* was isolated from a sinus wash-out and was identified as a unique strain of *P. aeruginosa* that was also cultured from cough swabs. Despite treatment, successful eradication from the paranasal sinuses was not achieved.

**Conclusions:**

Few reports have addressed the paranasal sinuses as a reservoir for lung infection in cystic fibrosis patients despite increased recognition of the need to investigate this niche. In this case, attempts at eradication of *P. aeruginosa* present in paranasal sinuses including oral and nebulised antimicrobials proved unsuccessful. However, detection of *P. aeruginosa* in the paranasal sinuses instigated antimicrobial treatment which may have contributed to prevention of migration to the lower airways. Our outcome provides additional insight and may indicate utility of nasal lavage or nasal endoscopy in paediatric cystic fibrosis patients’ annual review clinic visits.

## Background

The Cystic Fibrosis (CF) patient airway is defective in ciliary function; resulting, due to ineffective removal, in a mucus-rich environment [1] favouring growth of bacteria [2], including potential pathogens, such as, but not limited to, *Staphylococcus aureus*, *Haemophilus influenza* and *Pseudomonas aeruginosa* [1] associated with chronic infection [3], decreased lung function [4] and accelerated respiratory disease [5]. Consequently, risk of mortality for paediatric CF patients with lower airway infection by *P. aeruginosa* has been reported as almost three times higher than for those without [5].

There has been limited study of the paranasal sinuses as reservoirs for *P. aeruginosa* [6–11]. However, it is believed that due to poor mucociliary clearance in the paranasal sinuses and the lower airways, and physiological similarities between the membranes of the sinuses and the conductive bronchi, that *P. aeruginosa* migration from the sinuses to the lower airways may occur [6, 12]. Chronic *P. aeruginosa* infection is often preceded by a period of intermittent colonisation of the airways [8, 13], sometimes in the absence of symptoms of infection or immunological responses [14]. Adding additional complexity, in the context of managing CF patients successfully, the colonising *P. aeruginosa* may undergo substantial genetic adaptation and diversification into subpopulations with varying phenotypic traits (including nutient utilisation, growth rates, exopolysaccharide production and antimicrobial resistances) and, with regard to microbiological analysis, differing colony morphologies [1, 8, 15, 16].

The paranasal sinuses may become a focus for regular examination or antimicrobial treatment in CF patients, such as functional endoscopic sinus surgery promoted by Danish clincians [13, 17]. Similarly, the oral cavity has been identified as a potential reservoir for pathogenic bacteria in CF patients [18, 19], with failure to determine presence of pathogens potentially detrimental to CF patients. In this case, we focus on and describe intermittent *P. aeruginosa* colonisation of the oropharyngeal region of a paediatric patient and, supporting the rationale of the Danish group, the role of the paranasal sinuses specifically as reservoirs that contributed to our failure to eradicate the pathogen despite successive antimicrobial administration.

## Case presentation

Ethical approval and informed consent for this study was approved by the HSE Mid-Western Regional Hospital Research Ethics Committee. A 12 year old asymptomatic patient (homozygous for Phe508del) with good lung function (mean forced expiratory volume -FEV_1_) from March 2009 to June 2014: 91.6 % ± 1.46 %) and normal bronchoscopy with no bacterial growth from two bronchoalveloar lavages (BAL) (considered the ‘gold standard’ method for collection of samples from the lower airways [20, 21]), displayed intermittent chronic *P. aeruginosa* infection of the oropharyngeal region (or at least detection using the oropharyngeal swab) between September 2006 to September 2014. This caused the patient’s parents to persistently submit elevated numbers of cough swabs for microbiological investigation. Eradication regimens comprised nebulised (via both mouth and nose) and oral antimicrobials, which appeared to clear the oropharyngeal region of *P. aeruginosa*, albeit transiently, but ultimately proved ineffective.

*P. aeruginosa* was first isolated in August 2006 from a cough swab, when the patient was four years of age and, thereafter, isolated once each year for three subsequent years. Microbiological assessment was in accordance with *Health Protection Agency Standard Operating Procedure (HPA SOP) 57* guidelines. On each occasion that *P. aeruginosa* was identified, the patient underwent eradication regimens until three follow up monthly cough swabs were negative for growth. Samples obtained from the patient remained clear of *P. aeruginosa* from September 2008 until May 2010, when the annual recurrence pattern was re-established for three subsequent years. In December 2010, the detection of *P. aeruginosa* resulted in administration of ciprofloxacin (oral – 500 mg) and tobramycin (nebulised – 300 mg), both b.i.d. for 28 days. However, the frequency of *P. aeruginosa* colonisation increased from March 2013, evident approximately every three months until January 2014. Although the patient never exhibited symptoms of sinusitis, and although no general anaesthesia and involvement of an ear, nose and throat (ENT) surgeon (JF) was necessary, nevertheless, a bilateral antral washout was performed in Dec 2012 and Sept 2014 as the patient was unable to expectorate and the concept of the paranasal sinuses acting as a reservoir for *P. aeruginosa* was considered.

*P. aeruginosa* was isolated from the right antral washing; therefore, the patient underwent the same eradication regimen as before, supplemented by maintenance therapy of tobramycin (300 mg per 5 mL nebulised solution) and colistin (2 M IU) administered via a face mask that allowed the antimicrobials to pass through the nasal cavity. The patient was encouraged to breathe predominantly through the nose during the nebulisation sessions to ensure maximum administration to the nasal cavity. Following this extended treatment, between February and September 2014 cough swabs remained *P. aeruginosa*-free. In September 2014, the patient underwent repeat antral sinus washing, bronchoscopy and cough swab collection. *P. aeruginosa* was again isolated from the right antral washing. The lower airways remained clear of *Pseudomonas*.

*P. aeruginosa* isolates underwent molecular (Variable Number Tandem Repeat) and phenotypic characterisation (Table 1). Antimicrobial sensitivity profiles were generated for each isolate (from August 2006 until September 2014) based on CLSI and EUCAST guidelines [22, 23] (Table 2). VNTR analysis demonstrated that all isolates represented the same unique *P. aeruginosa* strain. Phenotypic analysis classified all except two isolates (September 2013 and 2014) as mucoid, a characteristic not seen in the majority (6 out of 7) of isolates previously. Isolates were proteolytic and siderophore producing (Table 1). All isolates were susceptible to tobramycin, ciprofloxacin and meropenem, while the majority (all but antral washing Sept 2014) were also susceptible to pipercillin/tazobactum (Table 2).

**Table 1:** Phenotypic profile of *Pseudomonas aeruginosa* isolates from a paediatric patient over n 8 year period

		Phenotypic classification			
Date specimen taken	Specimen cultured from (Location)	Gram staining + ve or -ve	Mucoid (+ or -)	Proteolytic (+/++/+++)	Siderophore producing (+/++/+++)
11/08/06^a^	Cough Swab	-	-		
23/08/07^c^	Cough Swab	-	-		
07/08/08^c,d^	Cough Swab	-	-		
05/05/10^b^	Cough Swab	-	-		
14/12/10	Cough Swab	-	+		
22/04/11^b^	Cough Swab	-	-		
10/04/12^b,c,d^	Cough Swab	-	-		
23/03/13	Cough Swab	-	+	+++	+++
06/06/13^c^	Cough Swab	-	+	++	+
30/09/13	Cough Swab	-	-	+++	++
20/12/13	Cough Swab	-	+	+	+
23/01/14^e^	Antral Washing	-	+	++	+++
11/09/14	Antral Washing	-	-	+	+

^a^First identification of *Pseudomonas aeruginosa*. ^b^ *P. aeruginosa* growth described as “scanty” by University Hospital Limerick microbiology laboratory. ^c^ *Staphylococcus aureus* also identified in addition to *P. aeruginosa*

^d^ *Haemophilus parainfluenzae* also identified in addition to *P. aeruginosa*. ^e^ *Pseudomonas fluorescens* also identified in addition to *P. aeruginosa*

Where + = low, ++ = intermediate, +++ = high

**Table 2:** Antimicrobial sensitivity profile for *Pseudomonas aeruginosa* from a paediatric patient over an 8 year period

	CLSI Guidelines								EUCAST Guidelines							
	Disk content (μg)	2006	2007	2008	2010		2011	2012	Disk content (μg)	2013				2014		
		11/8	23/8	6/8	5/5	14/12	22/4	10/4		26/3	6/6	30/9	20/12	23/1	23/1^a^	11/9^a^
Drug																
Ofloxacin	5	S	S	NT	NT	NT	NT	NT	0	NT	NT	NT	NT	NT	NT	NT
Pipercillin/ Tazobactum	100/10	S	S	S	S	S	S	S	36	S	S	S	S	S	R	S
Ceftazidime	30	S	S	S	S	S	S	S	10	S	S	S	S	S	S	S
Tobramycin	10	S	S	S	S	S	S	S	10	S	S	S	S	S	S	S
Ciprofloxacin	5	NT	S	NT	S	S	S	S	5	S	S	S	S	S	S	S
Amikacin	30	NT	S	NT	NT	NT	NT	NT	30	NT	NT	NT	NT	NT	NT	NT
Aztreoman	30	NT	S	NT	NT	NT	NT	S	30	S	I	I	I	I	I	I
Colistin (E-Test)	10	NT	S	NT	NT	NT	NT	NT	10	NT	NT	S	S	S	S	S
Imipenem	10	NT	S	NT	NT	NT	NT	NT	10	NT	NT	NT	NT	NT	NT	NT
Meropenem	10	NT	S	S	S	S	S	S	10	S	S	S	S	S	S	S

*S* Susceptible, *I* Intermediate, *R* Resistant and *NT* Not Tested

^a^ *P. aeruginosa* isolated from antral washing

## Conclusions

Our findings demonstrate that the paranasal sinuses acted as protected reservoirs for *P. aeruginosa* as verified by VNTR profiling, supporting previous (albeit few) reports [13, 17]. As with other researchers [16, 24, 25], we found indications of adaptation of the *P. aeruginosa* strain, whereby prior to March 2013, all but one *P. aeruginosa* isolate from our patient were classified (based on colony morphology) as non-mucoid, whereas on and after this date, four of six isolates were phenotypically mucoid (Table 1). Such mucoidy is due to an overproduction of polysaccharide alginate, which contributes to biofilm formation [26] and increased resistance to antimicrobials [27]. It is believed that a non-mucoid phenotype is most often associated with isolates from early infection while mucoid strains are more frequently associated with established infection [3] and, indeed, poorer patient prognosis [28]. In our study, the same isolates were identified as proteolytic (associated with damage to tissue [29]) and siderophore producing (linked to bacterial virulence [30, 31]) producing (Table 1), although no pattern of changing protease or siderophore activity was discernible.

Antimicrobials are used commonly in treatment of initial colonisation of *P. aeruginosa* to postpone chronic infection and respiratory dysfunction, often delivered orally (e.g., ciprofloxacin) or nebulised (e.g., tobramycin) [13, 32, 33]. In this case, analogous to the approach adopted successfully elsewhere [34, 35], additional maintenance therapy comprised nebulised tobramycin and colomycin on alternate months, for six months. However, in our case, this ultimately proved unsuccessful. Other groups have recognised the challenge of eradicating *P. aeruginosa* from the paranasal sinuses using existing approaches and have have begun to evaluate novel strategies such as inhalation of tobramycin using vibrating [36] or pulsating [37] aerosols or addition of hyaluronate to nasal sprays [38].

In our case, although the eradication regimen proved unsuccessful in decolonising the paranasal sinuses of *P. aeruginosa*, we believe that detection of the pathogen instigated antimicrobial treatment that may have contributed to prevention of migration to the lower airways and, therefore, preventing chronic infection of the lower airways. Our case supports the rationale for assessment of the paranasal sinuses as potential *P. aeruginosa* reservoirs in CF patients displaying intermittent colonisation patterns albeit that the currently available techniques are invasive, require general anaesthesia and prudent antimicrobial use as they may themselves, due to physical disruption, cause migration of bacteria to other airways.

## Consent

Written informed consent was obtained from the patient and his parents for publication of this case report. A copy of the written consent is available for review by the Editor of this journal.

## Abbreviations

CF: Cystic fibrosis
FEV: Forced expiratory volume
BAL: Bronchoalveolar lavage
HPA: Health Protection Agency
SOP: Standard operating procedure
ENT: Ear, nose, throat
CLSI: Clinical and Laboratory Standards Institute
EUCAST: European Committee on Antimicrobial Susceptibility Testing
VNTR: Variable number tandem repeat
